# 

**Published:** 1886-11-13

**Authors:** 


### THE HOSPITAL

An Institution, Family, and Parochial Journal of
Hospitals, Asylums, & all Agencies for the Care of the Sick, Criticism & News.

### CONTENTS.

**Medical Books and General Readers** - 105

**The Importance of Maintaining Medical Charities.** By H.R.H. The Duke of Cambridge - 106

**Hospital Worthies.**—Mrs. S. L. Gulson, founder of the Coventry Children's Hospital - - - - 106

**The Story of the Hospitals.** By One of the Public. —*continued* - - - - - - - - 109

**Words of Consolation.**—VII. The Privilege of Suffering - - - - - - - - - 110

**Flowers, Ferns, and Ward Decorations** - 111

**Notes and News.**—The Queen at Longmore Hospital.—Miscellaneous.—The late W. H. Vanderbilt.—New Clinical Hospital.—The Hospital Sunday Movement in Birmingham.—Munificent Bequests.—The Nottingham General Hospital.—Upton Lunatic Asylum.—Trained Nurses' Club.—West Ham Hospital Sustentation Fund.—Proposed Hospital for Bournemouth.—John Howard on Registration-fee Payments.—New Hospital at Hastings.—The Working Classes and the Hospitals, etc., etc. - 112

**The Quilter Prizes.**—Conditions, etc. - - - 115

**Annotations.**—Hospitals, Provident Dispensaries, and the Pay System.—Hospital Sunday and Individual Contributions.—Hospital Sunday Practices to be avoided. —The Asylums Board and Clinical Teaching - - 115

**Difficult Cases.**—The Case of Spinal Curvature - 117

**Incidents of Animal Life** - - - - 117

**Dolly in the Hospital** - - - - - 118

**Reviews and Notices.**—Practical Medicine.—Home Remedies - - - - - - - - 119

**In- and Out-Patients' Hospital Diary** - - 120

**The Children's Corner.**—Puzzles, Answers, etc. - vi

**The Hospital Charity Box.**—Prizes and Results - vi

